# Tick tock, tick tock: Mouse culture and tissue aging captured by an epigenetic clock

**DOI:** 10.1111/acel.13553

**Published:** 2022-02-01

**Authors:** Christopher Minteer, Marco Morselli, Margarita Meer, Jian Cao, Albert Higgins‐Chen, Sabine M. Lang, Matteo Pellegrini, Qin Yan, Morgan E. Levine

**Affiliations:** ^1^ Department of Pathology Yale School of Medicine New Haven Connecticut USA; ^2^ Department of Molecular, Cell, and Developmental Biology University of California Los Angeles Los Angeles California USA; ^3^ Rutgers Cancer Institute of New Jersey New Brunswick New Jersey USA

**Keywords:** aging, calorie restriction, DNA methylation, epigenome, in vitro techniques, longevity, oxidative stress, replicative senescence

## Abstract

Aging is associated with dramatic changes to DNA methylation (DNAm), although the causes and consequences of such alterations are unknown. Our ability to experimentally uncover mechanisms of epigenetic aging will be greatly enhanced by our ability to study and manipulate these changes using in vitro models. However, it remains unclear whether the changes elicited by cells in culture can serve as a model of what is observed in aging tissues in vivo. To test this, we serially passaged mouse embryonic fibroblasts (MEFs) and assessed changes in DNAm at each time point via reduced representation bisulfite sequencing. By developing a measure that tracked cellular aging in vitro, we tested whether it tracked physiological aging in various mouse tissues and whether anti‐aging interventions modulate this measure. Our measure, termed CultureAGE, was shown to strongly increase with age when examined in multiple tissues (liver, lung, kidney, blood, and adipose). As a control, we confirmed that the measure was not a marker of cellular senescence, suggesting that it reflects a distinct yet progressive cellular aging phenomena that can be induced in vitro. Furthermore, we demonstrated slower epigenetic aging in animals undergoing caloric restriction and a resetting of our measure in lung and kidney fibroblasts when re‐programmed to iPSCs. Enrichment and clustering analysis implicated EED and Polycomb group (PcG) factors as potentially important chromatin regulators in translational culture aging phenotypes. Overall, this study supports the concept that physiologically relevant aging changes can be induced in vitro and used to uncover mechanistic insights into epigenetic aging.

## Graphical abstract

We proposed a culture aging model to train a novel epigenetic age predictor, CultureAGE, and validated the measure for multi‐tissue physiological relevance in multiple aged mouse in vivo cohorts. We then investigated the inner workings of the clock and found culture epigenetic aging can be modulated via caloric restriction and reprogramming and is driven by CpGs enriched in Polycomb group factors.

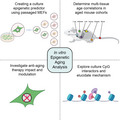

**Abbreviations**
BloodAGE: Blood epigenetic predictor
CH3: Methyl group
CpG: 5’—C—phosphate—G—3’
CR: Caloric restriction
CultureAGE: Culture epigenetic predictor
DNAm: DNA methylation
Giggle score: Chromatin regulator enrichment score
iPSCs: Induced pluripotent stem cells
LTK1: Large T antigen K1 mutant
MEFs: Mouse embryonic fibroblasts
PCA: Principal component analysis
PcGs: Polycomb group factors
PRC1: Polycomb repressive complex 1
PRC2: Polycomb repressive complex 2
RRBS: Reduced representation bisulfite sequencing
SASP: Senescence‐associated secretory phenotype
SA‐β‐gal: Senescence‐associated beta‐galactosidase
TF: Transcription factor
TSS: Transcription start site

## 1 INTRODUCTION

Aging is characterized by a progressive decline in cell, tissue, and organ integrity that manifests as age‐related diseases and ultimately death (Campisi et al., 2019). Telomere attrition, cellular senescence, DNA damage, stem cell exhaustion, and epigenetic modifications represent just a few molecular and cellular features of the aging process (Blasco, 2007; Oh et al., 2014; Tchkonia et al., 2010). While these hallmarks have been extensively investigated, their interactions, causes, and the resulting emergence that leads to the failure of the organism is not well characterized. Epigenetic alterations in aging—specifically alterations in DNA methylation (DNAm)—is a clear example of a hallmark which has been widely studied but lacks a conceptual mechanistic framework linking its causes and consequences to other hallmarks or physiological manifestations with aging.

DNA methylation refers to the addition of a methyl group (CH3) to a CpG dinucleotide (5’—C—phosphate—G—3’). In most cases, DNAm is associated with transcriptional repression via its effect on chromatin accessibility and is thought to control a number of cellular properties, including differentiation, replication, X‐inactivation, stress response, and genomic imprinting (Ferry et al., 2017; Izzo et al., 2020; Li et al., 1993; Riggs, 1975). Initially, de novo methyltransferases establish methylation patterns that are necessary for organismal development (Hata et al., 2002). These patterns are then modulated by maintenance methyltransferases over the course of the lifespan (Fuks et al., 2000). Subtle changes in DNAm can dramatically alter promoter function and distal regulatory elements (Aran et al., 2013). Changes in DNAm with aging were first reported more than three decades ago and now occupy a major field in aging research (Mays‐Hoopes, 1989). These changes paint a picture characterized by a gain of DNAm at gene promotors and loss of global DNAm, representing trends toward hypomethylation in intergenic regions associated with dispersed retrotransposons, heterochromatic DNA repeats, and endogenous retroviral elements. Given the predictability of these age‐related changes, researchers began applying machine learning techniques to develop age predictors from DNAm that could serve as biomarkers of aging. To date, these “epigenetic clocks” have been applied in a plethora of tissues across diverse mammalian species and are predictive of lifespan and health span above and beyond chronological age (Hannum et al., 2013; Horvath, 2013; Levine et al., 2018). However, the mechanistic underpinnings and drivers of epigenetic clocks are relatively unknown, limiting the conclusions that can be drawn.

Our lack of mechanistic understanding of epigenetic clocks likely stems from the fact that these models have been almost exclusively applied to in vivo and ex vivo blood and tissue samples in humans (and more recently in other mammals) for which experimental investigation is limited. Thus, we hypothesize that use of culture models coupled with physiologically relevant tissue samples may facilitate mechanistic discovery.

Culture aging has been extensively examined within the context of cellular biology, presenting a model to study mechanisms of epigenetic aging (Itahana et al., 2004). Since Hayflick proposed the theory now known as the Hayflick limit (Hayflick, 1965), many studies have contributed to characterizing exhaustive passaging, providing robust and well‐characterized culture models that can be used to determine the extent culture aging recapitulates physiological aging (Bork et al., 2010; Parrinello et al., 2003). However, none have applied systems‐level measures to directly demonstrate whether changes that can be induced in culture mimic what happens with aging in the organism. Thus, the aims of this paper were as follows: (i) to better characterize the culture aging phenomena by generating a clock based on DNA methylation changes in vitro, (ii) test whether such culture models of aging capture a physiologically relevant signal, and (iii) use this data as a first step toward elucidating mechanisms of aging. Overall, the results from this study set the foundation for using culture aging epigenetic models as a translational bridge to in vivo biomarker studies.

## 2 RESULTS

### 2.1 Developing a measure of culture aging using DNAm

**FIGURE 1:**
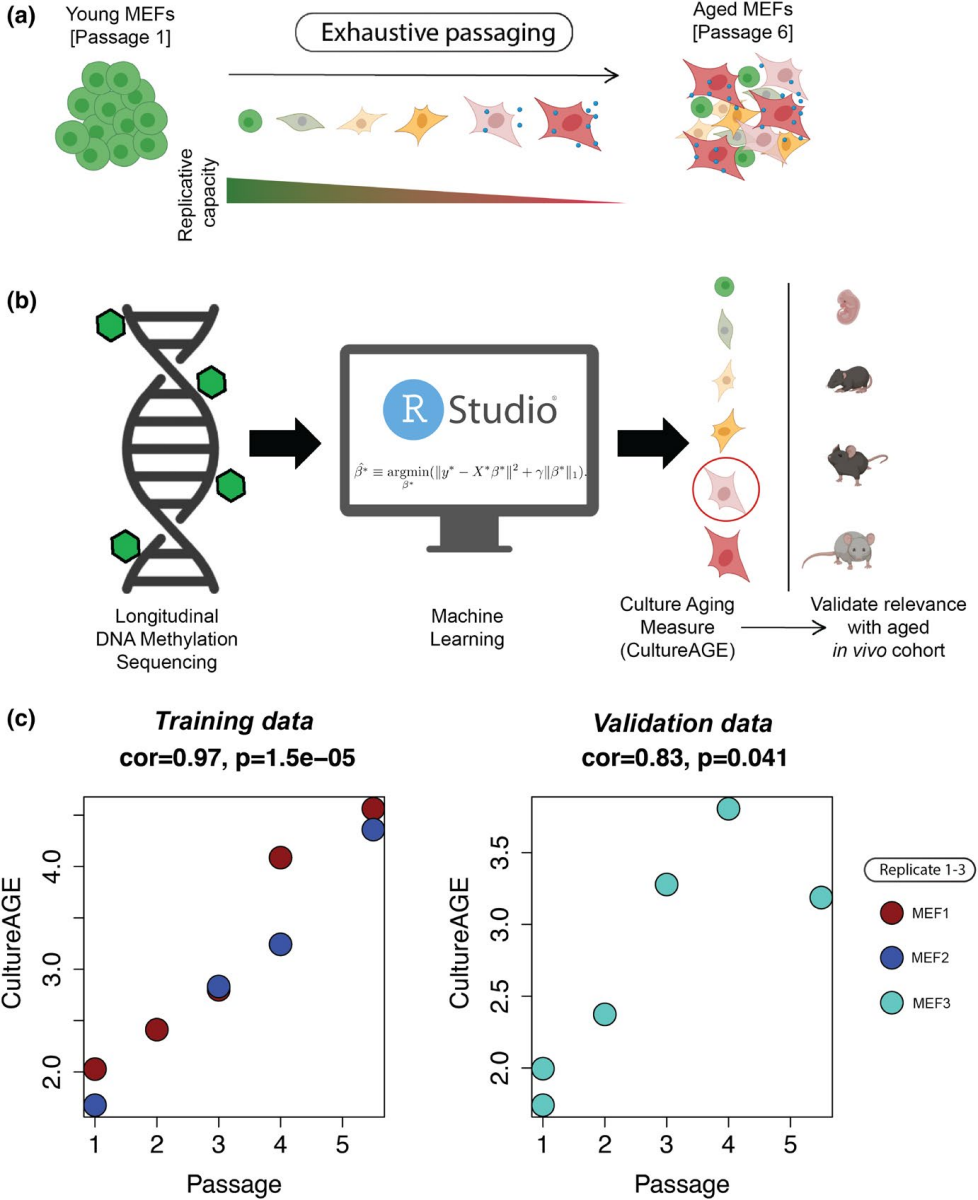
Development of a DNAm culture aging measure (CultureAGE) in mouse embryonic fibroblasts. (a) Schematic displaying exhaustive culturing of mouse embryonic fibroblasts under normoxia (20% O2) produces terminally arrested cellular states with progressively reduced replicative capacity. (b) Workflow demonstrating supervised machine learning computation approach (elastic net penalized regression) successfully produced a measure of culture aging from longitudinal reduced represented bisulfide sequencing (RRBS) DNA methylation data, where it was then was tested for physiological relevance in an aged in vivo cohort. (c) Training (MEF1 and MEF2) and validation (MEF3) cell lines used to develop CultureAGE. Red = MEF1, Blue = MEF2 and Turquoise = MEF3 replicates. Passage correlations and statistical significance were determined using Pearson correlations

To explore culture aging, understand its association with the methylome and determine the extent to which culture phenotypes recapitulate physiological aging, we derived a primary mouse embryonic fibroblast culture system that was exhaustively passaged to produce longitudinal DNAm samples (Figure 1a, Figure S1a–d). We selected mouse embryonic fibroblasts (MEFs) as our model, given their accelerated aging phenotype after relatively few passages (5–7) under normoxic (20%) conditions (Parrinello et al., 2003). This accelerated aging is hypothesized to occur from extrinsic factors, like oxygen toxicity, rather than intrinsic factors like telomere shortening (Itahana et al., 2004). It is also a distinct phenotype in contrast to MEFs grown under physiological conditions of 3% oxygen, which senesce at a much later passage. Given that genotoxic stress is known to modulate the methylome (Basenko et al., 2015; Colman et al., 2000; Liu et al., 1996), we reasoned that this model could enable us to capture the known murine sensitivity to oxidative damage using DNAm from serially passaged MEFs under normoxia.

DNA methylation was assessed at each passage in three biological replicates via reduced representation bisulfite sequencing (RRBS) with the goal of utilizing machine learning techniques to reduce the highly dimensional DNAm data into a single meaningful measure that increases as a function of time in culture (Figure 1b). The primary data used to train the culture measure, termed CultureAGE, were obtained from passages 1–6 of the culture MEF system. Of the three MEF cell lines, two were used in training (MEF 1 and 2) and the third (MEF3) was used for validation. In both cases, passages 5 and 6 were combined during sequencing (due to low individual DNA content) and designated as passage 5.5. Thus, our training data included samples at passage 1 (*N* = 2), passage 2 (*N* = 1), passage 3 (*N* = 2), passage 4 (*N* = 2), and passage 5.5 (*N* = 2). Initial principal component analysis (PCA) of training (*N* = 9) and validation (*N* = 6) MEFs revealed passage‐based trajectories in all replicates, suggesting the methylome is modulated as a function of time in culture (Figure S1e,f).

Prior to training CultureAGE, we sub‐selected common CpGs between our MEF data, Petkovich et al., 2017, and Thompson et al., 2018 to generate a list of 28,323 common CpG sites (Figure S2a). This was done so that our measure could be calculated in these external datasets to undergo a robust in vivo validation. Next, we conducted principal component analysis (PCA) on the 28k sub‐selected CpGs in our MEF data frame (*N* = 48). The initial PCA‐included some samples that were not explicitly analyzed, but reported passage number, so they were included to increase sample size. We previously found that combining PCA with elastic net yields more robust and reliable epigenetic age measures (Higgins‐Chen et al., 2021; Levine et al., 2020), and thus, we applied a similar strategy here. Elastic net penalized regression was used to generate a predictor of passage number, but rather than feeding in CpGs as has been traditionally done in epigenetic clock development, we used PCs as predictors in our model. The lambda penalty was chosen via 10‐fold cross‐validation and resulted in a model that included six PCs (PC2, PC4, PC6, PC8, PC9, and PC29) (Figure S2b–e). Overall, this measure is based on data from all 28,323 CpG sites, but is able to identify and combine the important patterns in genome‐wide DNAm to generate a single score, CultureAGE.

Our results showed that CultureAGE was highly correlated with passage number in both the training data (*r* = 0.97), and in our independent validation samples (*r* = 0.83), suggesting the marker is in fact progressively tracking with passage or time in culture (Figure 1c). In our training samples, we find that the measure shows a general linear increase. However, in the validation, there is a slight attenuation of the effect at the last passage. Given that we only have data on one sample at that passage, we cannot determine whether the non‐linearity is real, and follow‐up studies should increase power. One potential biological explanation is that there may be a deceleration at later cellular stages due to slowing in the growth rate from oxidative damage as cells approach or enter senescence.

### 2.2 Distinguishing senescence from epigenetic aging

**FIGURE 2:**
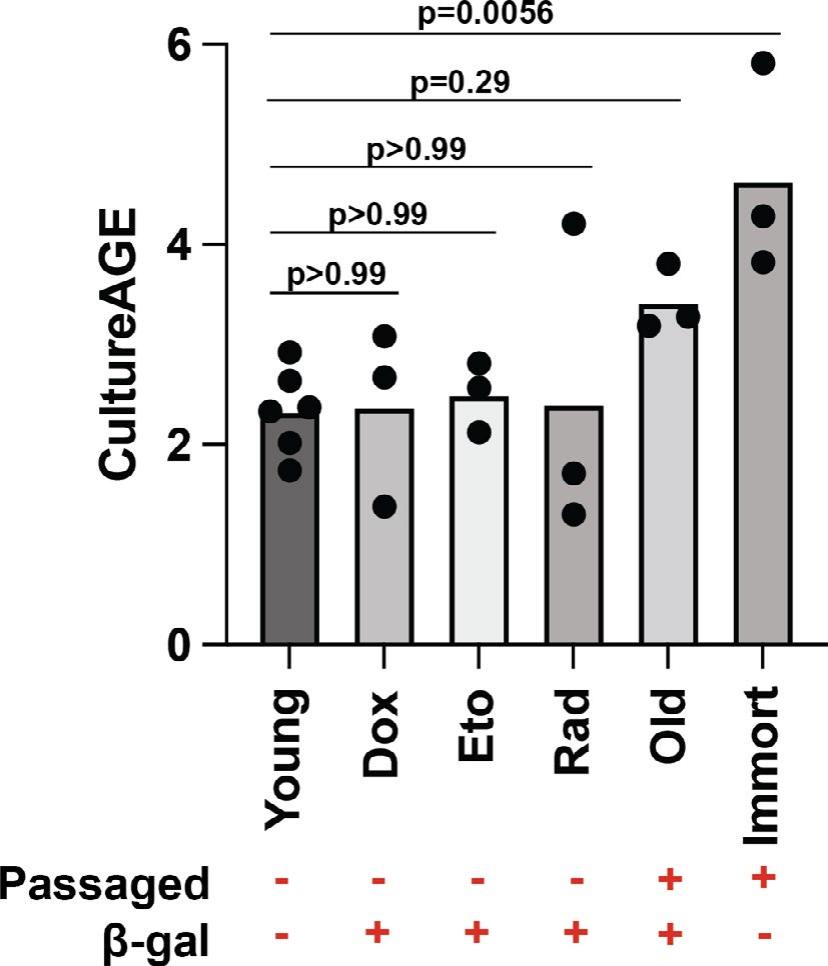
CultureAGE phenotype is independent of cellular senescence phenotype and requires replicative expansion. Boxplot displaying varying CultureAGE scores in young (untreated and DMSO, passage 1 or 2), passage‐independent (passage 2) senescence induction doxorubicin (1 µM), etoposide (12.5 µM), irradiation (10 gy), old (passage 3–5.5), and LTK1 immortalized cells (passage 5). Passaged label denotes cells were mitotically expanded, where β‐gal label establishes a binary senescence cutoff based on flow cytometry data in Figure S3. Statistical significance calculations were determined via one‐way ANOVA and multiple group comparisons

Replicative exhaustion in murine cells under normoxic (20% O2) conditions is a robust inducer of cellular senescence and we confirm in our study that MEFs arrest after 6 passages (Figure S1b–d). Based on this, we tested whether our epigenetic measure was as follows: (i) linked to senescence induction, likely as a result of chronic activity of a tumor repressor response to genotoxic stress, or (ii) reflects aging changes that are independent of senescence state, which may be overcome by immortalization (Figure 2). To test these questions, we induced senescence in a passage‐independent fashion using damaging dosages of irradiation (10 gy), doxorubicin (1 µM), and etoposide (12.5 µM). We show that each of these inducers elicits increased activity of SA‐β‐gal similar to levels exhibited in replicative senescence cells (Figure S3a–d); however, SA‐β‐gal levels are not related to CultureAGE (*r* = 0.062, *p* = 0.81). To further clarify if CultureAGE was capturing passage‐independent states, we transformed young MEFs with the known mouse immortalization agent, Large T antigen (LT) K1 mutant (LTK1) (Lin et al., 2011), and expanded the cells for 5 passages. Under the reduced p53 activity, the immortalized cells maintained high replicative states and demonstrated reduced SA‐β‐gal levels compared to passaged match controls (Figure S3e). Further, we found that immortalized cells showed acceleration in CultureAGE, suggesting that the DNAm changes captured progress or “tick” as a result of replicative events, not senescence status or other stress driven programs alone (*p* = 0.0056) (Figure 2).

#### 2.2.1 CultureAGE tracks and is correlated with multi‐tissue physiological aging programs

**FIGURE 3:**
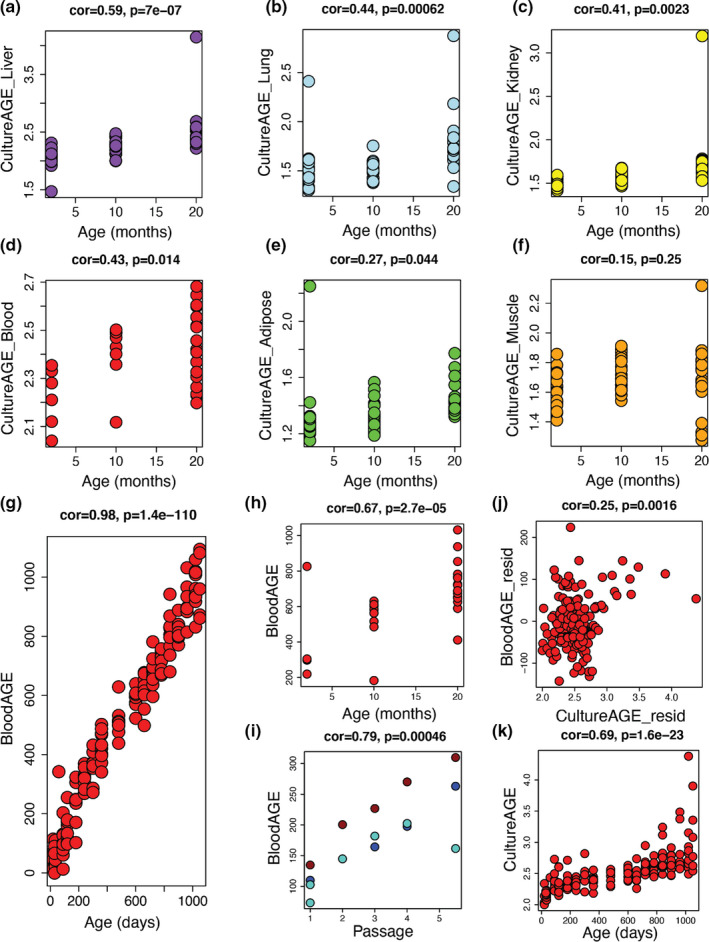
Multi‐tissue physiological aging is modeled by CultureAGE measure. CultureAGE score determined in liver (a), lung (b), kidney (c), blood (d), adipose (e), and muscle (f) tissue at 2, 10, and 20 months in aged C57BL/6J mice from Thompson et al., 2018. (g) BloodAGE epigenetic clock age association in blood training data from Petkovich et al., 2017. BloodAGE was trained in Petkovich et al., 2017 as a mouse age predictor using classical elastic net methodology, but with PCs as input variables, similar to CultureAGE. The final BloodAGE measure was constructed with 52 PCs and is validated using blood data from Thompson et al., 2018 in (h). (i) BloodAGE culture aging association in all MEF replicates used in CultureAGE training and validation. Red = MEF1, Blue = MEF2, and Turquoise = MEF3 replicates. (j) CultureAGE variance is associated with BloodAGE when residualizing by age in Petkovich blood data (age range, 20–1050 days). (k) CultureAGE measure in Petkovich blood data. Age correlations and statistical significance were determined using Pearson correlations

We tested whether these in vitro changes captured by CultureAGE mirror what is observed in aging tissues and cells in vivo, to determine whether CultureAGE is a valid aging biomarker. We applied our measure to in vivo multi‐tissue mouse DNAm data at three time points (ages 2, 10, and 20 months) from C57BL/6J mice from Thompson et al., 2018. CultureAGE significantly increases with age in five of the six tissues: liver (*r* = 0.59, *p* = 7.0e‐7), lung (*r* = 0.44, *p* = 0.00062), kidney (*r* = 0.41, *p* = 0.0023), blood (*r* = 0.43, *p* = 0.014), and adipose tissue (*r* = 0.27, *p* = 0.044) (Figure 3a–f). A moderate‐to‐low age increase was observed in skeletal muscle, although it was not significant (*r* = 0.15, *p* = 0.25) (Figure 3f).

#### 2.2.2 CultureAGE shares common epigenetic programs with ex vivo trained blood age estimator, BloodAGE

To further explore the physiological link of our culture aging theory, we compared the measure against a traditional ex vivo trained epigenetic clock measure, BloodAGE. Because RRBS data are sparse and few CpGs are consistently captured across experiments, we were unable to utilize previously developed mouse epigenetic clocks. Thus, we developed a novel BloodAGE clock using the same selected 28k CpGs used throughout the study. We trained the blood age predictor in a large blood dataset (age 20–1050 day C57BL/6J mice) from Petkovich et al., 2017 (cor = 0.98, *p* = 1.4e‐110) (Figure 3g), which we then validated in the blood dataset from Thompson et al., 2018 (cor = 0.67, *p* = 2.7e‐5) (Figure 3h), and confirmed it tracks with passage in the MEF data (cor = 0.79, *p* = 0.00046) (Figure 3i). Finally, we tested the overlap in signal (after residualizing for chronological age) between BloodAGE and CultureAGE in the blood aging data, which revealed a significant correlation (cor = 0.25, *p* = 0.0016), confirming CultureAGE is capturing similar signals to classically trained clocks (Figure 3j). Furthermore, CultureAGE demonstrates a strong positive association with age in the BloodAGE training data (*r* = 0.69, *p* = 1.6e‐23) (Figure 3k), and interestingly, some older mice demonstrated very high CultureAGE. Given that lymphoma is a common cause of death in aging mice, it is possible that CultureAGE reflects a predisposition to cancer (Haines et al., 2001).

### 2.3 Investigation into anti‐aging therapies confirms CultureAGE is modulated via caloric restriction and reprogramming

**FIGURE 4:**
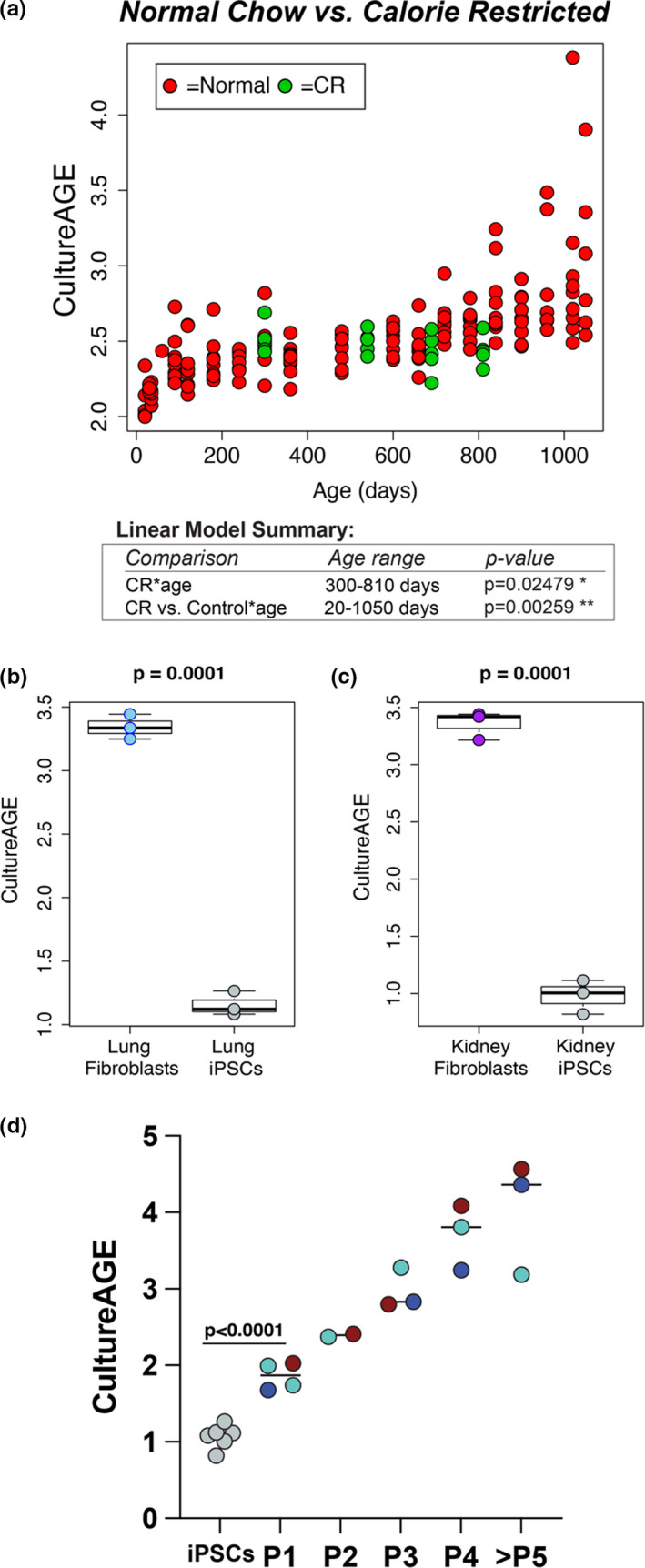
CultureAGE predicts naïve culture states in caloric restricted mice and re‐programmed fibroblasts. (a) Scatterplot demonstrating deceleration of culture aging in calorie‐restricted C57BL/6J mice from Petkovich et al., 2017, when comparing normal chow (20–1050 days) from calorie restriction (300–810 days) cohorts. Red samples represent normal chow diet and green samples calorically restricted diet. Calorie‐restricted mice began treatment at 14 weeks of age. Linear modeling demonstrates statistically significant deceleration in culture aging in CR samples (*p* = 0.02479) as well as significant modulation in CR‐treated mice compared to normal chow controls (*p* = 0.00259), when corrected by age. iPSC reprogramming in lung (b) and kidney (c) fibroblasts from Petkovich et al., 2017 demonstrates resetting of culture signature. (d) CultureAGE assessment of pooled lung and kidney re‐programmed iPSCs from (b and c) compared to MEF data, demonstrating reprogramming re‐sets and erases cellular states further than passage 1 MEFs (*p* < 0.0001). Red = MEF1, Blue = MEF2, and Turquoise = MEF3 replicates. Reprogramming and iPSC statistical significance calculations were determined via un‐paired two‐tailed *t* test

Using the Petkovich et al., 2017 data, we also found that CultureAGE was responsive to dietary intervention, such that calorically restricted (CR) mice exhibited significantly lower CultureAGE scores relative to controls (*p* = 0.00259), perhaps highlighting improved cellular maintenance and health from dietary intervention (Figure 4a). Finally, using the same dataset we showed that CultureAGE exhibits a decrease or resetting in lung (Figure 4b) and kidney fibroblasts (Figure 4c) upon reprogramming to induced pluripotent stem cells (iPSCs) (*p* = 0.0001). Specifically, the re‐programmed cells were reset to more youthful origins than even the passage 1 MEFs (*p* < 0.0001), suggesting culture aging established epigenetic networks are possible to completely reset upon reprogramming (Figure 4d).

### 2.4 Clustering analysis confirms culture aging exists in physiological context and highlights Polycomb group (PcG) factors as important culture aging regulators

**FIGURE 5:**
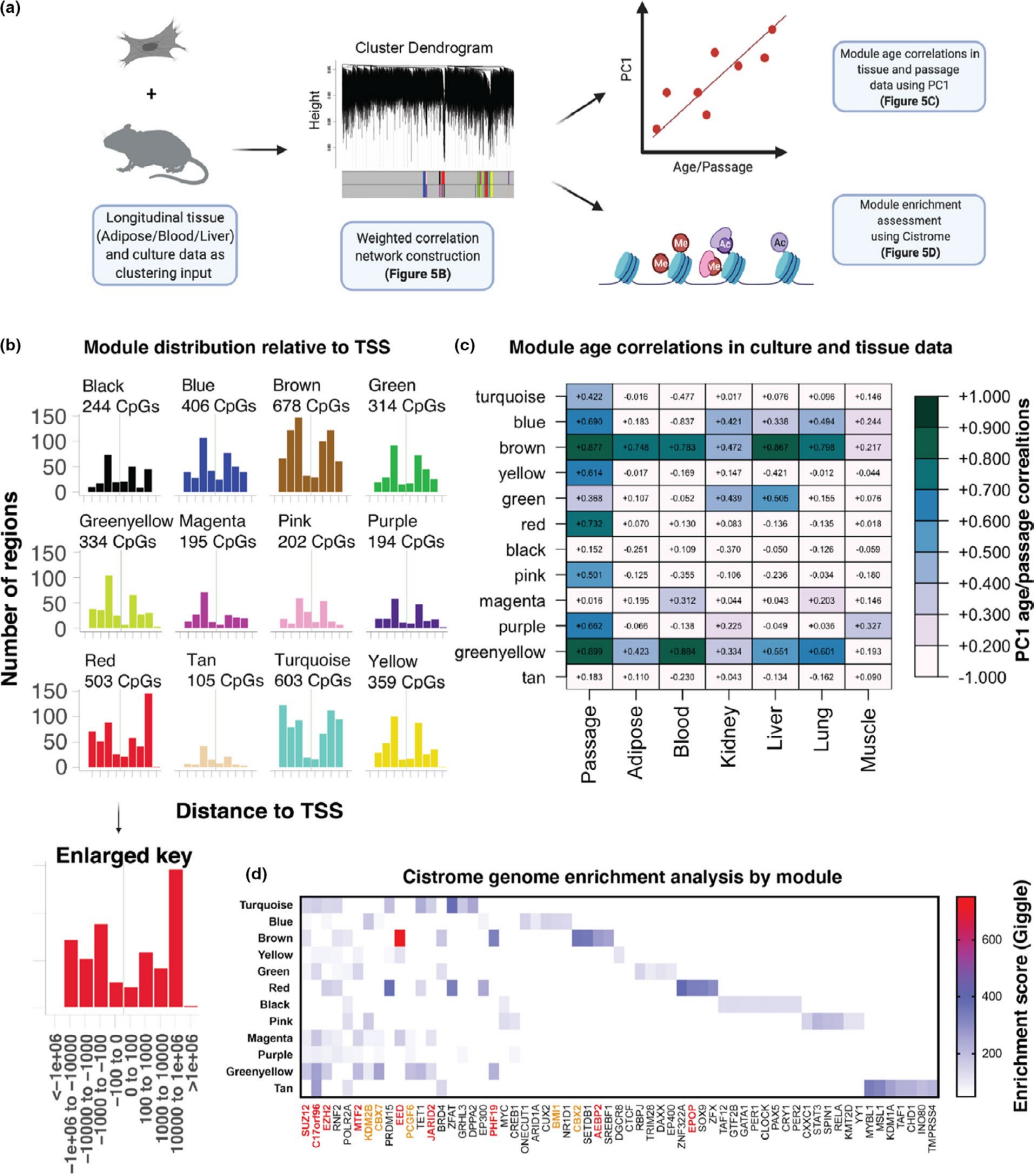
Clustering analysis confirms culture aging exists in physiological context and highlights Polycomb group (PcG) factors as important culture aging regulators. (a) Schematic outlining method of using longitudinal aging data (tissue + culturing) from Thompson et al., 2018 and the MEF1/MEF2 training data to cluster CpGs with WGCNA into distinct modules or ageotypes, which were then compared to in vitro passaging data and all tissues via principal component analysis and used to determine enriched genes using the Cistrome database. (b) Module distribution as determined by distance (per base pair) to transcription start site (TSS), generated using LolaWeb. Raw module CpGs were used to determine principal component correlations in (c), where kME selected CpGs were used to normalize enriched domains in (d), as further explained in Figure S4b. (c) PC1 correlations of longitudinal tissue and MEF passaging data by module. (d) Module genome enrichment analysis using Cistrome database from 100 CpG input selected by kME. Enriched genes were further normalized by randomly selecting 100 CpGs from the background 27,035 CpGs used to create the modules and correcting each enriched GSM_IDs Giggle score. Note, the enrichment analysis is displaying the average normalized enriched gene Giggle score (Top 10 displayed). Enriched genes are sorted by decreasing module frequency. Giggle score represents a rank of significance between genomic loci shared between query file and thousands of genome files from databases like ENCODE. Red genes = PRC2 complex or mediator, Orange genes = PRC1 complex or mediator, and Black genes = non‐Polycomb‐related genes

Given that CultureAGE is a composite measure stemming from multiple aspects or domains of DNAm changes, we hypothesized that some of the signal it encompasses may be physiologically relevant, while others may be culture artifacts or MEF‐specific phenotypes. For instance, we reasoned that supervised machine learning approaches, like elastic net, will prioritize strong signals in our culture models, despite whether they are physiologically relevant, limiting our ability to isolate important biological mechanisms. To address this, we applied consensus weighted gene correlation network analysis (WGCNA) to identify clusters (or modules) of highly co‐methylated sites that are conserved across both in vivo (Petkovich et al., 2017; Thompson et al., 2018) and in vitro data (Figure 5a, Figure S4a). We used 27,035 CpGs as the input, which excluded beta values of 0 from the original 28,323 overlapped CpGs. We identified 12 CpG modules, ranging in size from 105 to 678 CpGs. Most modules showed bimodal distribution in relation to distance from transcription start sites (TSS), with many showing peaks at ±100–1000 bp (Figure 5b).

Next, we estimated module eigengenes that capture the main signal from each module and tested their associations with passage number (in vitro MEF data) and age (in vivo tissue data). Eigengenes were calculated as PC1 estimated from the in vitro data and then applied as validation to the in vivo data (Figure 5c). Using these values, we observed several modules that appear to be artifacts of in vitro aging (turquoise/yellow/red/pink/purple), such that they showed progression with passage number in MEFs, but did not track with age in tissues. However, two modules (brown and greenyellow) stood out as being shared between culture and tissue aging. For instance, the brown module was strongly correlated with passage number (*r* = 0.88), as well as age in liver (*r* = 0.87), lung (*r* = 0.80), blood (*r* = 0.78), and adipose (*r* = 0.75). It was also moderately correlated with age in kidney (*r* = 0.47) and weakly correlated with age in skeletal muscle (*r* = 0.22). The greenyellow module exhibited strong correlations with both passage number in vitro (*r* = 0.90) and age in blood (*r* = 0.88), while showing moderate age correlations with lung (*r* = 0.60), liver (*r* = 0.55), adipose (*r* = 0.42), and kidney (*r* = 0.33), and a weak correlation with age in skeletal muscle (*r* = 0.19). As a comparison, we applied the CultureAGE PCloadings and coefficients to the module CpGs to determine the relative CpG contributions by module based on the initial selection criteria established by CultureAGE (Figure S5a). We confirm our hypothesis that certain artifactual drivers are present in CultureAGE (turquoise/red), but also highlight that major physiological signals do indeed exist, with the brown module comprising nearly 17% of the normalized CultureAGE score, compared to the average of 8.3%. Additionally, the average CpG contribution across all PCs demonstrates the majority of the brown module CpGs are enriched when compared to random chance or artifactual modules like red and pink, and poorly correlating modules like black (Figure S5b).

Finally, to garner more biological insight into potential mechanisms at work in conserved modules, we assessed genome enrichment of transcription factor (TF) binding motifs and chromatin regulators using the Cistrome database. This was done by comparing each module by TF and chromatin regulator enrichment score (Giggle score). The Giggle score represents a rank of significance between genomic loci shared between query file and thousands of genome files from databases like ENCODE. Given that scores tend to increase for lists with a greater number of input genomic locations (and thus would be biased by module size), we normalized each module prior to determining the enrichment score so that only 100 CpG locations were being assessed for each module. For instance, we selected the top 100 CpGs with the highest kME values in a given module. kME is estimated as the correlation between CpG values and the module eigengene and can be used to infer connectivity or identify “hubs” of a module. For the background CpGs, we selected 100 CpGs from the 27,035 CpG background at random and used the background Giggle score to blank any hit overlap from the modules. The Giggle score threshold was the actual value below which scores were blanked. The final 100 input CpGs for each module are reported by genomic partition distribution (Figure S4b) and scatterplots of each raw Cistrome distribution are reported by module (Figure S4c). We compared the average normalized enriched gene Giggle score from each module to determine the most enriched gene regulators.

The Cistrome analysis (Figure 5d) reveals that Polycomb repressive complex 1 and 2 (PRC1 and PRC2) networks are highly enriched in translational modules (brown and greenyellow), highlighting Polycomb group proteins (PcGs) as key epigenetic regulators in both culture and physiological aging. Nearly all of the top hits for greenyellow (9/10) occurred in PcGs, the highest Giggle enrichment score occurred in EED (PRC2 components) for the brown module, and the only shared hit between greenyellow and brown was PHF19, a PRC2 recruitment zinc‐finger domain. Finally, we conducted Cistrome analysis on the top module CpG contributors to CultureAGE and found that 8/10 of the hits were PcG components, including EED and PHF19 (Figure S5c). Altogether, our data suggest PcGs regulate physiologically relevant culture aging phenotypes.

## 3 DISCUSSION

Given that well‐characterized culture systems exist (Parrinello et al., 2003), we aimed to classify potential epigenetic drivers of culture aging and determine if such changes model physiological aging in various tissues and biofluids. We rationalized that with the widespread use of culture models throughout biology and medicine, many fields would greatly benefit from clarifying the underlying epigenetic phenotypes that exist in culture and whether relevant markers of cellular dysfunction can be trained for use in accelerating mechanistic and drug development discoveries.

By exhaustively passaging primary MEFs under normoxic conditions (20% O2), we trained a DNAm predictor of passage number (time in culture), called CultureAGE, and demonstrate that it not only accurately tracks passage number (Figure 1c) but also strongly correlates with age in multiple tissues (liver, lung, kidney, blood, and adipose) in vivo (Figure 3a–f), captures similar signals to a blood trained epigenetic clock (BloodAGE) (Figure 3g–j), is modifiable by dietary intervention (Figure 4a), and exhibits resetting upon reprogramming to pluripotency (Figure 4b–d). Interestingly, skeletal muscle was the only tissue examined where CultureAGE did not correlate with age (Figure 3b), which may reflect that skeletal muscle remains mostly postmitotic in adulthood or that muscle cells are multinucleated. The link between proliferation and CultureAGE was also observed when comparing the other tissue types. For example, we observed differences in both age correlation/slope, and in the absolute scores when comparing tissues. Overall, samples from liver and blood appeared to exhibit the highest values (Figure 3a,d), which may reflect the higher proliferative capacity of cells in these samples or the renewable nature of both hepatocytes and blood cells, perhaps suggesting that lifetime damage is somehow cataloged by the methylome. This is also substantiated by the observations that epigenetic aging is not linear with time (Levine et al., 2018). For instance, previous epigenetic clocks have been shown to increase rapidly during development and then decelerate after full maturity. We were able to observe this same trend in our data. We found that CultureAGE exhibited a sigmoidal function with age, characterized by accelerated aging during development, a slower and more linear increase after about 150 days, and exponential increases at late life (Figure 3k).

Despite the evidence of a relationship between replication and epigenetic aging, our data suggests that this is independent of senescence accumulation. For instance, we showed that drug and irradiation‐induced senescence in MEFs was not associated with changes in CultureAGE (Figure 2, Figure S3a–c). Further, our results demonstrate CultureAGE does not predict cellular senescence when compared by senescence status (assessed via Beta‐galactosidase activity) for pooled passage‐independent senescence (irradiation and drug induced) and replicative senescence samples (cor = 0.062, *p* = 0.81) (Figure S3d). Follow‐up studies should explore senescence‐associated secretory phenotypes (SASP) in the context of acute culture stress, in order to build upon our conclusions using β‐gal as a marker of cellular senescence. To fully conclude CultureAGE was not driven by senescence states, we tested cells immortalized with LTK1 and still found acceleration in the rate of CultureAGE, despite suppressed senescence signal (Figure 2, Figure S3e). Cells immortalized via LTK1 have inactive p53, leading to reduced senescence accumulation compared to passage‐matched controls (Figure S3e). Importantly, p53 and/or Rb inactivation are sufficient for murine fibroblast immortalization (Lin et al., 2011). The maintained cellular progression captured by CultureAGE in old immortalized (non‐senescent) samples may be attributed to the underlying tumor suppression inactivation occurring from LTK1 transformation, allowing continued mitotic progression and damage accumulation without cell cycle arrest and senescence perturbations.

The potential links between epigenetic aging, replication, and genotoxic stress may also explain the age‐related increase in cancer susceptibility, particularly among highly proliferative tissues and cells. For instance, we and others have previously reported that epigenetic age changes are observed at increasing rates in tumors and/or the normal (or non‐afflicted) tissues of individuals with cancer. We reason that the epigenetic changes captured by measures like CultureAGE may underlie susceptibility to spontaneous transformation or oncogenicity (Levine et al., 2019). Cells that eventually evade senescence from mutational events may promote oncogenic states, allowing continued mitotic events and increased damage accumulation, as a function of cell turnover. In moving forward, it will be critical to utilize future in vitro experiments to determine the mechanisms driving epigenetic changes as a function of either mitotic rate (replication “ticking”) and/or prolonged exposure to genotoxic stress. Our laboratory has already extended these mouse culture aging results to human culture models, where we recently showed exhaustively passaged astrocytes capture epigenetic aging trajectories when modeled using established clocks (Higgins‐Chen et al., 2021).

While substantial work has gone into developing biomarkers than enable researchers to track aging changes in vivo and in vitro, the ultimate goal is to develop measures that are also modifiable to intervention. Using DNAm assessed in blood, we reported the effects of two promising interventions in aging—caloric restriction (CR) and cellular reprogramming. Our results suggested that CultureAGE showed strong response to CR when assessed in blood (Figure 4a). Multiple studies suggest that CR acts by reducing DNA damage accumulation and mutations that progress with age (Heydari et al., 2007), where others show CR downregulates key growth hubs like the insulin/IGF1 pathway (Li et al., 2011). Importantly, IGF1 is a growth factor that stimulates cell proliferation and can promote cancer via inhibition of apoptosis (Kari et al., 1999). Interestingly, CR, without malnutrition, has also been shown to reduce cancer incidence and progression in mice (Chaix et al., 2014). Our results suggest that CR could be acting via the epigenome to regulate DNA damage maintenance by slowing cellular turnover and thus damaged states, or perhaps from enhanced DNA repair. Additionally, our results showed that the longer mice underwent CR, the more they diverged from normal controls on the basis of CultureAGE. This could suggest that prolonged CR does not simply reverse, or retard epigenetic aging momentarily, but actually decelerates the rate of change with age.

We also report renewal in lung and kidney fibroblasts indicative of naïve culture states following reprogramming to iPSCs, supporting the conclusion that CultureAGE cannot only be slowed, but actually reversed (Figure 4b–d). For instance, both lung and kidney fibroblasts derived from 10‐week‐old mice and broadly passaged were predicted to be equivalent to passage 3–4 cells, while all iPSC derivatives were reset to more youthful origins than the passage 1 MEF data (*p* < 0.0001) (Figure 4d). This suggests that the major epigenetic changes acquired during culturing and/or tissue aging (Sturm et al., 2019) can be reset to some extent. It is unlikely DNA damage and the resulting genome instability is reversible, thus we propose that CultureAGE may be capturing transient epigenetic programs that control survival, proliferation, and cellular maintenance.

In the current study, we also tested whether we could distinguish different “types” of DNAm changes in our data, using a network‐based clustering approach. Our results clearly demonstrate that in vitro DNAm changes captured some modules that were not physiologically relevant, suggesting that they may be reflective of culturing or MEF‐specific artifacts. In contrast, CpGs in two modules (brown and greenyellow) appear to capture a common epigenetic aging phenotype that is established in both physiological and culture aging context (Figure 5a–c). We found evidence that PcG factors, including both PRC1 and PRC2, are key factors in physiologically relevant culture aging (Figure 5d). Additionally, upon applying the PCloading conditions to the modules, we confirm major physiological signals do in fact exist in CultureAGE (Figure S5a,b), and that the top CpG contributors are also enriched in PcG factors (Figure S5c). It is well established that the tri‐methylated histone H3 at lysine 27 (H3K27me3) mark denotes transcriptional silencing with PRC2 involved in early development and PRC1 later during aging as the more active maintenance factor (Cao et al., 2002). The catalytic subunit of PRC2, EZH2, is routinely overexpressed in oncogenesis (Kim & Roberts, 2016), promoting uncontrolled cell growth, as many repressed downstream genes of H3K27me3 are tumor suppressors (Bracken et al., 2007), but the role of PRC2 and its domains are conflicted in aging. In certain species and cell types, EZH2 mutations reduce H3K27me3 and confer longevity (Ma et al., 2018), although in others reduction of H3K27me3 is associated with aging (Maures et al., 2011). The relationship between the catalytic subunit (EZH2) and its co‐factors SUZ12, EED, RbAp48, and AEBP2, which are highly involved with allosteric recognition and binding of substrates like *S*‐Adenosyl methionine (SAM), is multi‐factorial, with many opportunities for perturbations. As an example, multiple studies demonstrate EZH2, SUZ12, and EED are essential components for proper functioning, but RbAp48 and AEBP2 are not (Cao & Zhang, 2004). Our reported translational modules (brown/greenyellow) further support the notion that PcGs are important aging factors, and our culture aging system may be useful for testing hypotheses about PcG roles in aging.

In conclusion, we report a novel mouse epigenetic measure of culture aging, termed CultureAGE, that is able to model epigenetic changes observed in multiple in vivo tissues. CultureAGE is independent of senescent state, and instead appears to capture progressive cellular changes that may confer susceptibility to senescence and/or tumorigenesis. We also provide evidence for potential modifiability in the form of deceleration as a function of CR or reprogramming. Finally, DNAm changes may be functionally related to Polycomb group (PcG) factors like EED. Overall, this study demonstrates that physiologically relevant DNAm changes can be modeled in vitro, which in the future can be used to interrogate mechanisms involved in epigenetic aging and/or facilitate in vivo aging discoveries.

## 4 METHODS

### 4.1 Experimental

#### 4.1.1 Mouse embryonic fibroblast extraction

Mouse embryonic fibroblasts were harvested at day 12.5 of gestation. Two females were used. From the first female, nine embryos were sacrificed and split into three cell lines, MEF1–3 from the second female, 10 embryos were sacrificed and split into three cell lines, MEF4–6.

Extraction was achieved by separating embryos into separate wells in a 6‐well dish using PBS, removing inner embryo and using forceps to carefully remove limbs, head, and internal organs from dorsal region. The dorsal region was then cut and trypsinized for 10 min at 37°C. To quench reaction cells were transferred to a 15 ml falcon tube and spun for 3 min at 300g, then supernatant was aspirated and resuspended with 10 ml DMEM. P0 cells were split once to expand cell number prior to freezing. Approximately 2 ml of cells were incubated overnight with 8 ml DMEM and following growth were trypsinized and either passaged for experiments or stored at −80°C in DMEM/DMSO (90:10).

#### 4.1.2 Replicative passaging and cell culture

Cells were split/passaged six times, where flow cytometry/confocal microscopy and RRBS sequencing were conducted at each passage.

Cells were split according to the following seeding density—p100 – 0.5 × 10^6^ cells, p60 – 0.25 × 10^6^ cells, and six well – 0.125 × 10^6^ cells—and were counted using an Invitrogen countess and cell counting chamber slide with trypan blue. For media, we used DMEM +10% FBS +1% PENSTREP. Note, later passaged cells had a lower plating efficiency when inspected visually 24 h after seeding, thus we used a cell scraper prior to transfer otherwise senescent cells remained attached to the dish. Cells were split at approximately 95% confluence which occurred around 3–4 days in P1–3 and 5–8 days in P4–6.

#### 4.1.3 Plasmid transfection

LTK1 (Immortalization) and empty vector (pBABE) plasmids were described previously (Lin et al., 2011). Briefly, Phoenix Amphotropic cells were used to grow virus as described previously (Pear et al., 1993) and puromycin (0.5 µg/µl) and blasticidin (2 µg/µl) were used for selection.

#### 4.1.4 Beta‐galactosidase flow cytometry and confocal microscopy

To conduct beta‐galactosidase flow cytometry, approximately 0.25 × 10^6^ cells were seeded into p60 dishes and pre‐treatment was conducted approximately 16 h after seeding. Cells were first pre‐treated with Bafilomycin A1 (Selleckchem: S1413, 622.83 g/mol, 100 µM stock). Existing DMEM was aspirated, then cells were washed with PBS and replaced with treated Bafilomycin A1 DMEM for 30 min at a final concentration of 100 nM. Following Bafilomycin A1 pre‐treatment to normalize lysosome activity, C12FDG (Invitrogen: D2893, 853.92 g/mol, 10 mM stock) was added directly to the existing media for 90 min at a final concentration of 20 µM. Note, due to light sensitivity, exchange was conducted in a dark environment.

For determining beta‐galactosidase activity via flow cytometry, treated cells were trypsinized (1 ml‐p60) for 5 min at 37°C and then quenched using 3 ml DMEM. Note, cells were completely detached using a cell scraper prior to transfer otherwise senescent cells remained attached to the dish. After thorough resuspension, cells were transferred directly to a filter top tube and spun for 3 min at 1200 rpm. Supernatant was aspirated, and cells were resuspended in 100 µl PBS and immediately assayed using a 488 nM laser on a StratedigmS1000 benchtop flow cytometer. Fluorescence intensity was normalized and baselined using an unstained sample. FlowJo (10.6.1) was used to analyze data. Beta‐galactosidase activity/senescence activity was determined as LogFITC treated geometric mean/control geometric mean after normalizing to untreated control.

For determining beta‐galactosidase activity via confocal microscopy, cells were split into 12 well dishes with a glass cover slide at the bottom of each well. Following Bafilomycin A1 and C12FDG treatment, media was aspirated, and cells were washed with PSB 3×, fixed with 4% PFA/PBS (10 min), followed by 2× PSB washes and then counter stained with DAPI and mounted onto coverslips. Fixed cells were immediately imaged at 4×, 10×, and 40× resolution using a Keyence confocal cytometer.

#### 4.1.5 Senescence induction

We induced senescence using previously established conditions (Tchkonia et al., 2010). In brief, MEFs were thawed and allow to expand for one passage, then split to a normalized seeding density of 0.25 × 10^6^ cell/p60 and 0.125 × 10^6^ cells/6‐well and treatment was conducted for 5 days. Note, senescence induction experiments were conducted at passage 2. Doxorubicin (Sigma: D1515, 1 µM), Paclitaxel (Sigma: T7402, 50 nM), and Etoposide (Sigma: E1383, 12.5 µM) were all dosed into DMEM when the cells were split and media was not replaced for the duration of the 5‐day treatment. We irradiated cells (10 Gy) using cesium irradiation and collected these cells after 5 days as well.

#### 4.1.6 DNA preparation and quantification

DNA was extracted from selected samples prior to RRBS sequencing using a Qiagen DNeasy Blood and Tissue extraction kit (69504). Note, samples were treated with proteinase K and RNAse A and eluted with 200 µl elution buffer. Following final elution, DNA was verified using nanodrop (Thermo Scientific). Spin concentration was used as necessary with low DNA content samples. Prior to library preparation, we used a qubit fluorometer (Thermo Scientific) to quantify the extracted genomic DNA. All samples were assigned a single‐blinded code and randomized for library preparation and sequencing to control for any batch errors.

#### 4.1.7 Library preparation and reduced representation bisulfide sequencing

Library preparation was conducted using EZ DNA Methylation RRBS Library Prep Kit (Zymo: D5461), according to manufacturer's recommendations. Randomized and pooled samples were sequenced on four Illumina NovaSeq6000 SP lanes (100 bases single‐end mode). Note, each lane produced more than 400 M reads.

### 4.2 Statistical analysis

#### 4.2.1 Data preprocessing

FastQC (v0.11.8) was used to assess the quality of the raw reads and adapter‐trimmed reads (cutadapt, version 2.5). Reads were mapped to the GRCm38 RRBS genome using BSBolt v0.1.2 (https://github.com/NuttyLogic/BSBolt) (Farrell et al., 2021). Methylation was called and the CpG methylation matrix was assembled for CpG sites common to all samples and covered by more than 10 reads. The final matrix consisted of 466,359 CpG sites.

#### 4.2.2 Training and validation of DNAmCULTURE

R was the primary platform used for statistical analysis (Version 3.6.2). After selecting overlapped CpGs between training (in vitro) and all validation studies (in vivo), PCA (without scaling) was conducted in the training sample. The initial PCA was conducted on *N* = 48 MEF samples, all with reported passage number between 1 and 6. Note, some samples were not analyzed in this report. Briefly, *N* = 9 passaged (Passage 1–6) samples were used as the culture training samples for the elastic net regression selection of PCs. The outcome was 6 PCs, each with a PCloading for all 28,323 CpGs, then a specific coefficient for each PC, resulting in the predictor of passage number, called CultureAGE. Lambda penalty represented the value with lowest mean‐squared error, selected via 10‐fold cross‐validation (Figure S2b,c). Further details on PC‐trained DNAm measures can be found from our previous reports (Higgins‐Chen et al., 2021; Levine et al., 2020).

To validate the measure, PCs were estimated in independent MEF passaged samples that were not included in elastic net selection (MEF3) and external datasets (in vivo) using the loading from the training sample. These PCs were then incorporated into the selected elastic net model (Figures 1, 3, and 4). Pearson correlations were used to assess associations between CultureAGE and (1) passage number in both the training and validation sample, and (2) age in multi‐tissue in vivo samples. One‐way ANOVA multiple group comparisons were used for analyze senescence statistical significance. Two‐tailed *t* tests were used to compare significance in iPSC reprogramming and in MEF4 validation. To test for associations with CR, OLS regression was used that included age, CR, and an interaction term (age*CR).

#### 4.2.3 WGCNA and module construction

Consensus WGCNA (Langfelder & Horvath, 2008) was conducted using four input datasets—MEF training samples (replicates 1 and 2), and the Thompson et al. data for blood, liver, and adipose. The remaining Thompson et al. data (kidney, lung, and muscle) were deliberately excluded from WGCNA so as to have a true validation. In total, we used 27,035 CpGs as the input, which excluded beta values of 0 from the original 28,323 overlapped CpGs. Adjacency was estimated for each dataset based on biweight midcorrelations and negative correlations were treated as unconnected in the network (signed network). Adjacencies were then converted to Topological Overlap Matrices (TOMs) and combined into a single consensus TOM, such that overlap for each CpG pair was designated as the minimum dissimilarity score across the four individual TOMs. Hierarchical clustering was then conducted with the following parameters: deepSplit = 1, cutHeight = 0.95, minClusterSize = 50, and distance = 1‐consensus TOM, method = average. This resulted in a network with *n* = 16 modules. Given that similar modules can often be split by WGCNA, we next tested whether modules should be merged. This was done by estimating module eigengenes and then assessing dissimilarity between modules. Using a cut height of 0.4, the 16 modules were merged into 13 that served as our final modules for all remaining analyses.

One feature of WGCNA is the ability to estimate module eigengenes, which serve as single quantitative value meant to represent the core signal of a whole module—that can consist of tens to thousands of individual variables. Typically, PC1 from PCA run on all variables in a module is used to represent the module eigengene. However, the traditional WGCNA package estimates this separately for all dataset meaning that the eigengenes may not be based on the same equations across datasets (variables can have different loadings). This may cause a bias in results and make validation less straight forward. To overcome this, we estimated PC1 for each module using the MEF training data and then applied these loading to all other datasets, including those used in WGCNA and thus that were held‐out. Finally, we tested whether the module eigengene values were associated with either passage number (MEF data) or age (multi‐tissue data).

For calculating the module CpG contributions to CultureAGE, we applied the PCloading and coefficients to each module CpG and determined a CpG contribution score as the fold increase above a random event. More specifically, we summed the cumulative contribution per PC (across all 28,323 CpGs) and determined the average CpG contribution (or random chance) by baselining the score by 28,323 events (or the original CpGs used to calculate the PCs). Note, the absolute value of each PCloading was used. We then compared the PCloading sum across each PC (PC2, PC4, PC5, PC8, PC9, and PC29) by every module selected CpG and determined contribution as [PCloading*coefficient of Module CpG / PCloading*coefficient of average CpG]. For example, CpG contribution = 1 means the selected CpG site is not specifically selected over random chance, but CpG contribution >1 means the CultureAGE measure is selecting the CpG site to drive the score. The raw CpG contributions are plotted in Figure S5a, and the average across all PCs is plotted in Figure S5b. Finally, we normalized each module contribution by the number of CpGs in each module, which resulted in a normalized weight that we calculated as a percentage of the total module CpGs (*N* = 4137 CpGs) to produce a final normalized % contribution (Figure S5a).

#### 4.2.4 Cistrome genome enrichment analysis

We used the Cistrome gene analysis tool kit (http://dbtoolkit.cistrome.org/) to determine enriched genes. We selected the top 1 k hits and used the mm10 reference. The outcome of the enrichment analysis was reported as a Giggle score, which is a rank of genome significance between the input file and thousands of genome files from databases like ENCODE. It is important to note that Cistrome is constantly updating genome files, thus the enrichment analysis was conducted at the same time. Additionally, we selected 100 CpGs from each module using kME to select the most central 100 CpGs. Sub‐selected CpGs are reported via genomic partition in Figure S4b. For selecting the background 100 CpGs, we randomly selected the 100 CpGs from the cohort of 27,035 CpGs. For Giggle score reporting, we plotted the raw giggle score of each resulting module query, although any file (GSM_ID) that was also a background hit was corrected using the formula; GSM_ID_Hit‐GSM_ID_Background = GSM_ID_Actual. Note, when the background GSM_ID was not present there was no correction. We report raw giggle scores in a scatterplot format in Figure S4b and the average corrected values (Top 10 genes) in Figure 5d. For calculating the top CpG contributor (>5) enriched domains, we conducted a similar analysis, except 118 CpGs were used in both the >5 region and in background. All values are reported in Figure S5c.

Genomic partitioning and CpG locations were determined using LolaWeb (http://lolaweb.databio.org/).

## CONFLICT OF INTEREST

No competing interests to report.

## AUTHOR CONTRIBUTIONS

Contribution is based on authorship.

## Supporting information

Figure S1

Figure S2

Figure S3

Figure S4

Figure S5

## Data Availability

The data that support the findings of this study are available from the corresponding author upon reasonable request.
