# Supplementary material for: Tick tock, tick tock: Mouse culture and tissue aging captured by an epigenetic clock
Source: Aging Cell. 2022 Feb 1;21(2):e13553. doi: 10.1111/acel.13553 (PMC8844113; doi:10.1111/acel.13553)
Supplement: Supplementary file 1 — Figure S1 [file ACEL-21-e13553-s003.docx]

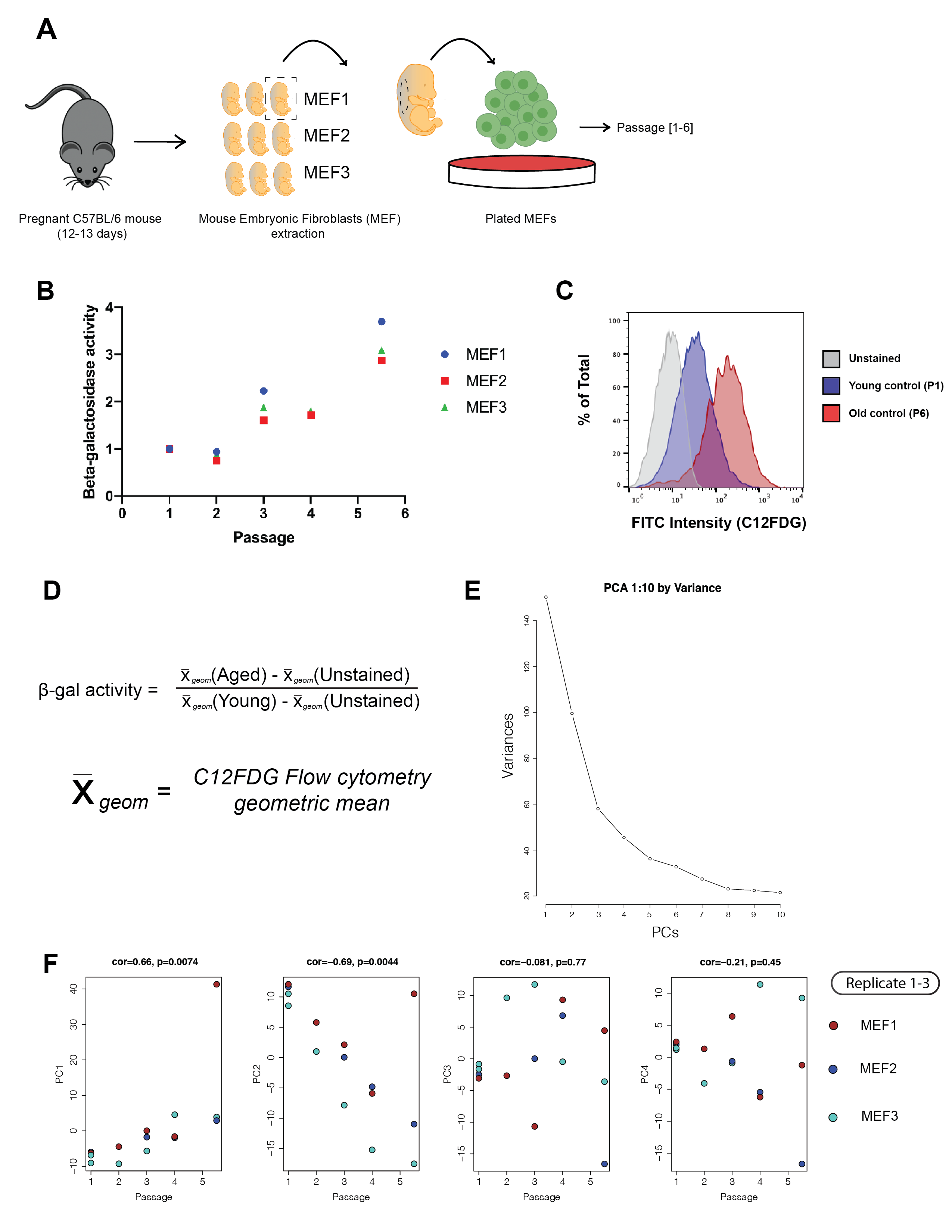


**Supplemental Figure 1: Mouse embryonic fibroblast extraction, passaging and validation.** (A) Schematic demonstrating MEF extraction, illustrating embryos were dorsally derived from 12.5-day gestation C57BL/6 mice, then passaged 6x. Note that each biological replicate was composed of 3 embryos. (B) Plot of flow cytometry data demonstrating increased Beta-galactosidase (β-gal) activity with passage, measured by FITC fluorescence from C12FDG and normalizing geometric mean with negative samples (unstained) and dividing crude fluorescence by young control (Passage 1). (C) Representative flow cytometry plot demonstrating older MEFs have greater beta-galactosidase activity. (D) β-gal activity calculation. (E) Plot measuring PCA components [PC1-10] from MEF1-3 (passages 1-6) as a function of variance in non-selected original 466k CpG sites. (F) Principal component analysis, trained from non-selected 466k CpG sites, of all MEF cell lines used for training (MEF1-2) and validating (MEF3) CultureAGE, confirming each replicate captured similar trajectories. Red=MEF1, Blue=MEF2 and Turquoise=MEF3 replicates.
