# Supplementary material for: Tick tock, tick tock: Mouse culture and tissue aging captured by an epigenetic clock
Source: Aging Cell. 2022 Feb 1;21(2):e13553. doi: 10.1111/acel.13553 (PMC8844113; doi:10.1111/acel.13553)
Supplement: Supplementary file 2 — Figure S2 [file ACEL-21-e13553-s001.docx]

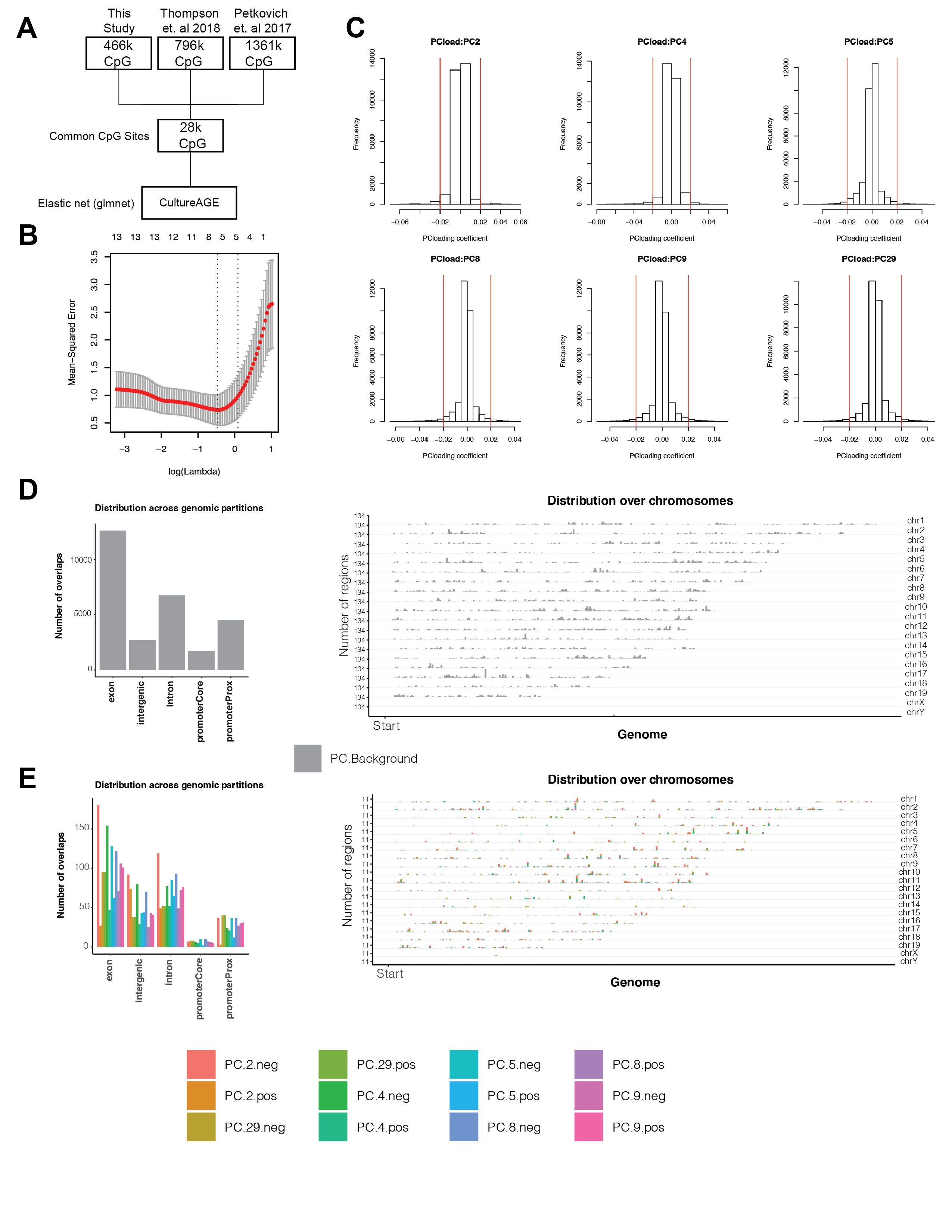


**Supplemental Figure 2: CultureAGE construction, PC loading and CpG distribution.** (A) Common CpGs (28,323) between MEF experimental data, Petkovich et. al 2017 and Thompson et. al 2018. (B) Elastic net penalized regression plot generating lambda minimum for selecting PCs (PC2, PC4, PC5, PC8, PC9 and PC29) for CultureAGE. (C) Histogram of loaded PCs in CultureAGE, plotted by PC loading coefficient and frequency of total 28,323 CpGs in measure. Red abline represents 0.02 cutoff used to determine CpG drivers of CultureAGE. PCA from N=48 samples (All passaged MEF samples with varying experimental perturbations) was used to feed into elastic net for selecting PCs. (D) CpG distribution across chromosomes and genomic partitioning of raw 28,323 CpGs used in CultureAGE, generated by LolaWeb. (E) CpG distribution across chromosomes and genomic partitioning of CpG drivers (N=3087), as determined by 0.02 loading coefficient cutoff, also generated by LolaWeb.
