# Supplementary material for: Tick tock, tick tock: Mouse culture and tissue aging captured by an epigenetic clock
Source: Aging Cell. 2022 Feb 1;21(2):e13553. doi: 10.1111/acel.13553 (PMC8844113; doi:10.1111/acel.13553)
Supplement: Supplementary file 3 — Figure S3 [file ACEL-21-e13553-s005.docx]

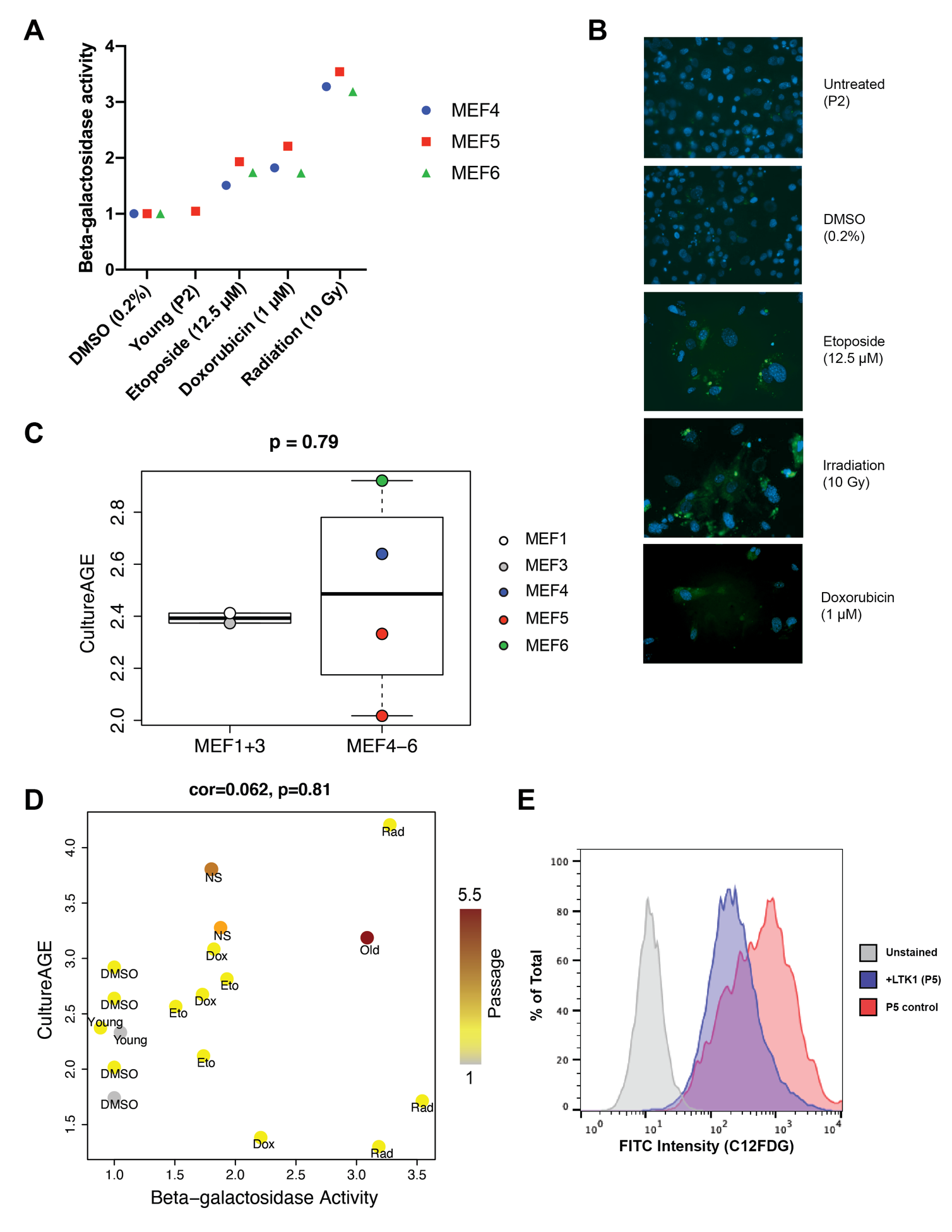


**Supplemental Figure 3: Passage independent senescence induction, MEF technical replicate and immortalization validation.** (A) Beta-galactosidase activity measured in MEF4-6 with various damaging and control conditions. β-gal activity was determined by LogFITC fluorescence from C12FDG flow cytometry and normalizing geometric mean with negative samples (unstained) and dividing crude fluorescence by DMSO or young control, depending on experiment. β-gal activity calculations are further explained in Supplemental Figure 1D. (B) Representative confocal microscopy images (40X) using C12FDG (green) and counterstained with DAPI (blue), confirming senescence is achieved from irradiation and drug treatment (doxorubicin and etoposide). (C) CultureAGE measured in all MEF replicates for passage 2 samples under control conditions (either Young untreated or DMSO), demonstrating no significant inherent variation exist between replicates. Statistical significance calculations were determined via un-paired two-tailed t-test. (D) CultureAGE measured in irradiation and drug induced senescent samples against DMSO, young (passage 2) and passaged controls (passage 3-5.5). Note, Dox=doxorubicin (1 µM), Eto=etoposide (12.5 µM), Rad=irradiation (10 gy), NS=near senescent (passage 3 and 4) and old=passage 5.5. Treatment occurred for 5 days. Senescence induction procedures are outlined in the methods. Passage independent experiments were conducted in MEF4-6 cell lines, which were validated by comparing passage 2 DNAmCULTURE score to MEF1 and MEF3 samples in (C). β-gal correlations and statistical significance was determined using Pearson correlations. (E) Representative flow cytometry plot displaying reduced β-gal activity (C12FDG) in cells transformed and immortalized with LTK1. Note, both immortalized and control cells were passaged 5 times. LTK1 transfection experiments were conducted in MEF1-3 replicates.
