# Supplementary material for: Tick tock, tick tock: Mouse culture and tissue aging captured by an epigenetic clock
Source: Aging Cell. 2022 Feb 1;21(2):e13553. doi: 10.1111/acel.13553 (PMC8844113; doi:10.1111/acel.13553)
Supplement: Supplementary file 4 — Figure S4 [file ACEL-21-e13553-s004.docx]

**
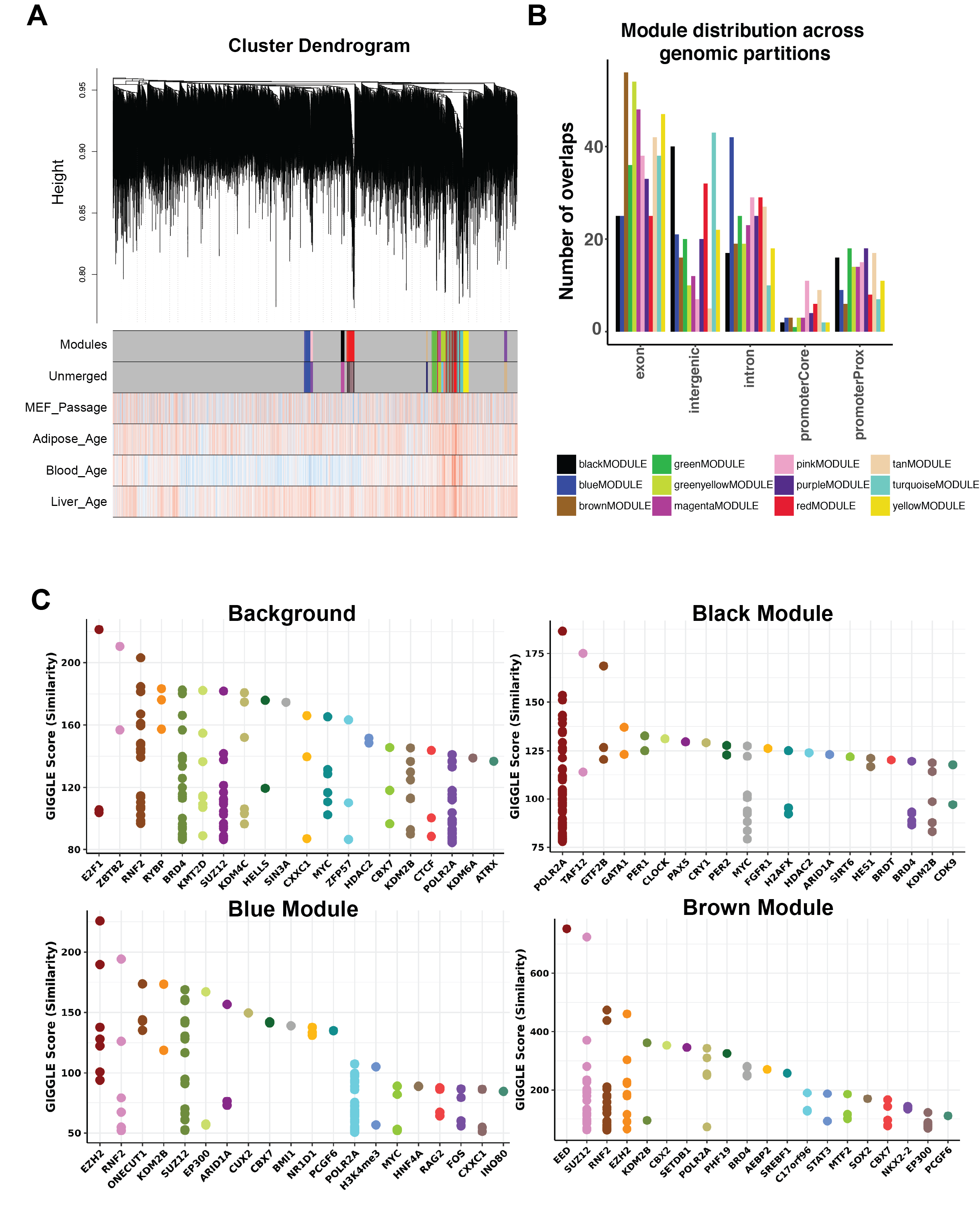
**

**
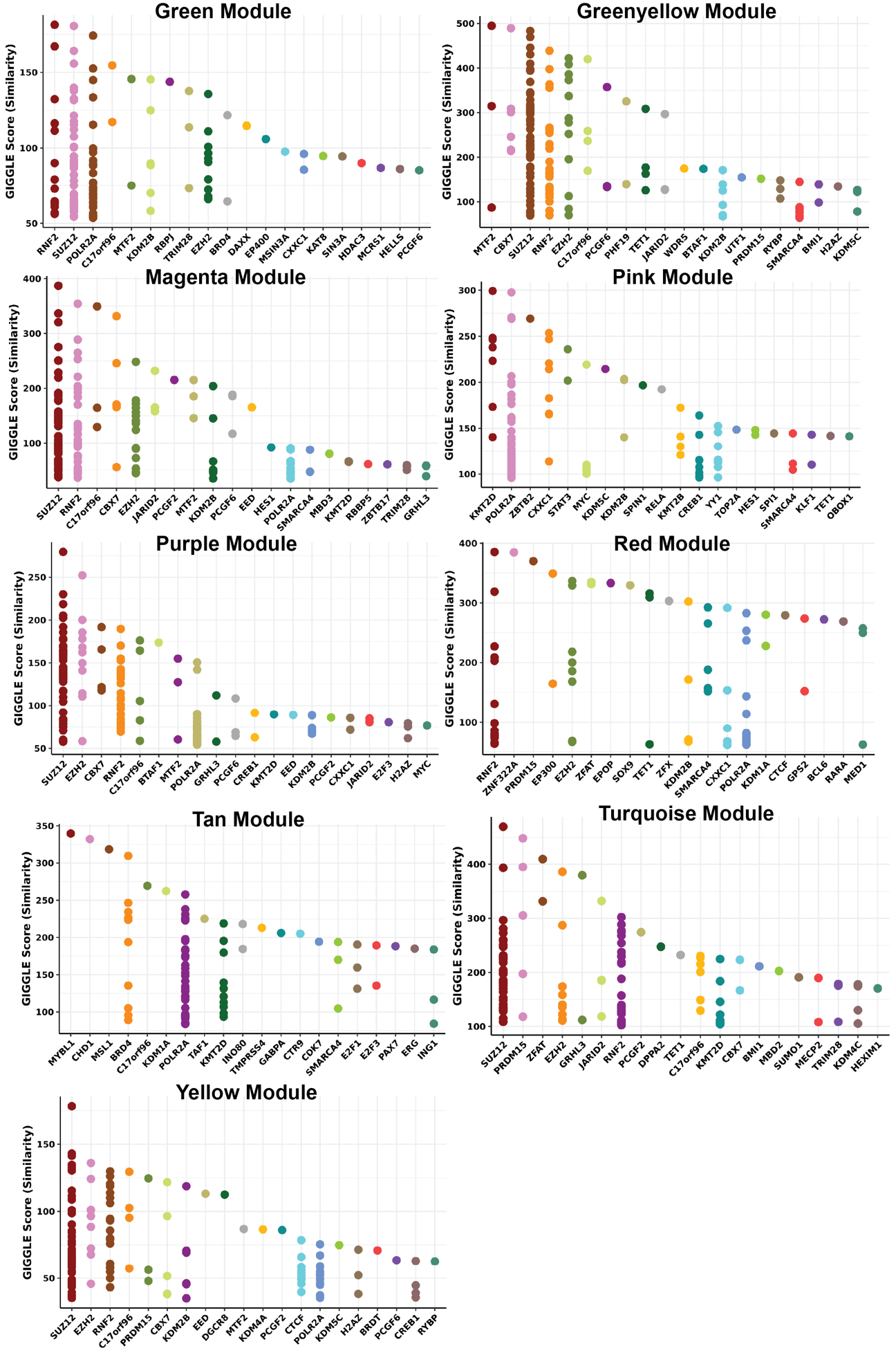
**

**Supplemental Figure 4: Network construction and Cistrome enrichment analysis.** (A) Cluster dendrogram demonstrating 12 modules and age/passage associations with culture, adipose, blood and liver input data. (B) Genomic partition (generated by LolaWeb) of top 100 CpGs, as determined by the most central CpGs by kME, of the 12 selected WGCNA modules. kME selected CpGs were used to normalize enriched domains in (C). (C) Scatterplots of top 20 enriched genes in each module, as determined by Cistrome, prior to any background baselining. Note, the top 100 CpGs, as determined by kME, were used as the input for each query in order to cross compare modules by Giggle score. 100 CpGs were selected at random from the 27,035 background CpGs that were used as the input for clustering analysis. Giggle score represents a rank of significance between genomic loci shared between query file and thousands of genome files from databases like ENCODE.
