# Supplementary material for: Tick tock, tick tock: Mouse culture and tissue aging captured by an epigenetic clock
Source: Aging Cell. 2022 Feb 1;21(2):e13553. doi: 10.1111/acel.13553 (PMC8844113; doi:10.1111/acel.13553)
Supplement: Supplementary file 5 — Figure S5 [file ACEL-21-e13553-s002.docx]

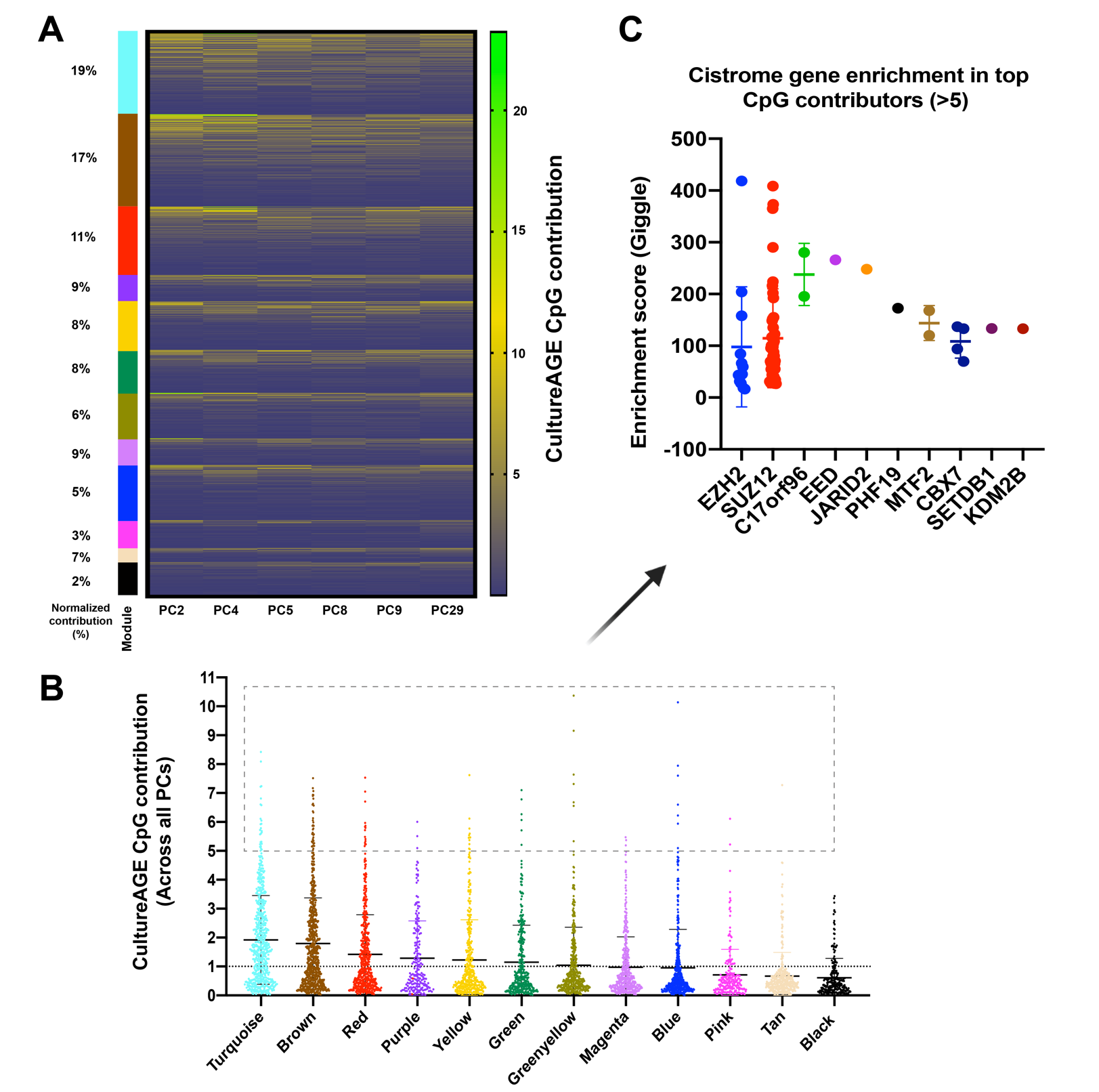


**Supplemental Figure 5: Generalized module contributions to PCloading scores from CultureAGE confirms physiological modules are positively selected and PcG factors are enriched in top CpG contributors.** (A) Heatmap of all module CpGs (N=4137) displaying PCloading transformed contribution, measured by fold increase compared to random event. For example, CpG contribution=1 means the selected CpG site is not specifically selected over random chance, but CpG contribution>1 means the CultureAGE measure is selecting the CpG site to drive the score. The normalized contribution % is the total weight each module contributes to the overall CultureAGE score, when limiting the selected CpGs to the 4137 module CpGs and normalizing by number of CpGs per module. Note, the original CultureAGE measure includes PCloading data on all 28,373 CpGs, where the module CpGs make up 14.61% of the total CpGs, but the module CpGs together make up 19.48% of the clock score, meaning overall the clock is positively selecting module CpGs. Finally, each PCloading score was transformed by the elastic net selected coefficient for each PC to produce an accurate picture of CultureAGE contribution. (B) Average CpG contribution across all PCs (PC2, PC4, PC5, PC8, PC9 and PC29), displayed by module. (C) Cistrome genome enrichment analysis of top 118 CpG contributors (>5) from (B). 118 CpGs were selected at random from the 4137 module CpGs and were used to normalize each hit. Again, Giggle score represents a rank of significance between genomic loci shared between query file and thousands of genome files from databases like ENCODE.
